# What we need is health system transformation and not health system strengthening for universal health coverage to work: Perspectives from a National Health Insurance pilot site in South Africa

**DOI:** 10.4102/safp.v62i1.5079

**Published:** 2020-09-03

**Authors:** Janet Michel, Brigit Obrist, Till Bärnighausen, Fabrizio Tediosi, Di McIntyre, David Evans, Marcel Tanner

**Affiliations:** 1 Swiss Tropical and Public Health Institute, Basel, Switzerland; 2 Department of Epidemiology and Public Health, Faculty of Science, University of Basel, Basel, Switzerland; 3 Department of Global Health, Harvard T.H. Chan School of Public Health, Boston, United States; 4 Institute of Public Health, University of Heidelberg, Heidelberg, Germany; 5 Health Economics Unit, University of Cape Town, Cape Town, South Africa; 6 World Bank, Washington, D.C., United States; 7 Department of Social Sciences, Faculty of Humanities and Social Sciences, University of Basel, Basel, Switzerland

**Keywords:** universal health coverage, health systems in transition, health systems no longer fit for purpose, health systems transformation versus health systems strengthening, policy implementation

## Abstract

**Background:**

Globally, universal health coverage (UHC) has gained traction as a major health priority. In 2011, South Africa embarked on a UHC journey to ensure that everyone has access to quality healthcare services without suffering financial impoverishment. National Health Insurance (NHI) and primary healthcare (PHC) re-engineering were two vehicles chosen to reach UHC over a 14-year period (2012–2026). The first phase of health system strengthening (HSS) initiatives to improve the quality of health services in the public sector began in 2012. These HSS initiatives are still being carried out by the Department of Health in conjunction with other partners.

**Methods:**

A qualitative case study design utilising a theory of change (TOC) approach was employed. Data were collected from key informants (*n* = 71) during three phases: 2011–2012 (contextual mapping), 2013–2014 (Phase 1) and 2015 (Phase 2). In-depth face-to-face interviews were conducted with participants using a TOC interview guide, adapted for each phase. All interviews were audio-recorded and transcribed verbatim. An iterative, inductive and deductive data analysis approach was utilised. Transcripts were coded with the aid of MAXQDA 2018.

**Results:**

Six broad themes emerged: make PHC work, transform policy development, transform policy implementation, establish public–private partnerships, transform systems and processes and adopt a systems lens.

**Conclusion:**

A third great transition seems to be sweeping the globe, changing how health systems are organised. Actors in our study have identified this need also. Health system transformation rather than strengthening, they say, is needed to make UHC a reality. Who is listening?

## Introduction

### Key messages

- Strong health systems are key to achieve universal health coverage (UHC). Policymakers at provincial level and implementing actors at district, sub-district and facility levels view the way the current health system in South Africa is designed as a hindrance to National Health Insurance (NHI) success and ultimately UHC.
- A third great transition seems to be sweeping the globe, changing how healthcare is financed and how health systems are organised; hence, actors are calling for health system transformation rather than health system strengthening to achieve UHC.
- Universal health coverage is an opportunity but not a guarantee for progress: getting things right now can have big pay-offs later, but letting things go wrong initially can be highly problematic and costly.

### Background

Universal health coverage has gained traction as a major health priority in many countries.^1^ There is also recognition of social determinants of health in contributing towards a long healthy life for all.^2^ Good health is an essential and indispensable prerequisite for poverty reduction, sustained economic growth and socio-economic development.^2^ In 2011, South Africa embarked on a UHC journey to ensure that everyone has access to quality healthcare services without suffering financial impoverishment.^2^ Multiple epidemics, powerful historical and social forces such as vast income inequalities, unemployment, poverty, racial and gender discrimination, the migrant labour system and extreme violence shaped the current health system,^2^ making it two tiered, public and private, based on socio-economic status, with one for the poor and the other for the rich. Poor management of public services threatens to further widen these disparities.^3^ Over 80% of South Africans have no health insurance, heaping the burden onto the public health system. The two-tiered system is unsustainable, destructive, very costly and highly hospicentric.^2^ Many South Africans remain impoverished with inferior access to healthcare except care for human immunodeficiency virus/acquired immunodeficiency syndrome (HIV/AIDS).^3^ The public health system faces a myriad of challenges, including a quadruple burden of disease and an increasing number of patients in need of antiretroviral therapy (ART) whilst simultaneously caring for millions of people already on ART.^4^ Human resources shortages^2,5^ and underperforming institutions, as a result of poor management, underfunding and deteriorating infrastructure, further compound the challenges.^2^

South Africa is in the midst of multiple interconnected social, economic, epidemiologic, demographic, technological, institutional and environmental transitions. These changes are impacting health and well-being as well as the capacity of health systems to respond.^3,4^ Prior to the 21st century, patients had an episodic relationship with the health system that was dictated by acute care needs.^6^ This service delivery approach is now incongruent with the current morbidity trends, which demand regular, continuous care.^6^ An upsurge of non-communicable diseases, other lifelong treatments and ageing populations have given rise to multi-morbidities and chronicity, necessitating care that is proactive rather than reactive; comprehensive and continuous, rather than episodic; and disease specific, founded on lasting patient–provider relationships rather than incidental provider-led care.^6^

Health system strengthening (HSS) involves initiating activities in the different components of a health system, and another way is to use the six World Health Organization (WHO)-HSS building blocks: human resources; health finance; health governance; health information; medical products, vaccines and technology; and service delivery.^7^ Two vehicles chosen by South Africa to achieve UHC are NHI and primary healthcare (PHC) re-engineering. Health system strengthening is a component of the South African NHI initiatives being rolled out in three phases, between 2012 and 2025.

Phase 1 (2012–2016) focused on quality improvement in the public sector. The Office of Health Standards Compliance (OHSC) was set up, audits of public health facilities aimed at improving quality were completed^8^ and PHC re-engineering teams were appointed.

Phase 2 (2017–2021) focuses on the development of NHI legislation (the NHI Bill was presented to Parliament for approval in 2019), aimed at establishing institutions needed for a fully functional NHI fund. This phase also entails purchasing personal healthcare services for vulnerable groups, such as children, women, people with disability and the elderly.

Phase 3 (2022–2025) will focus on ensuring that NHI is fully functional.^9^

Piloting HSS in South African pilot districts was foreseen for the 2012–2016 period. These HSS initiatives, which are the focus of this study, have been and are still being carried out by the Department of Health (DoH) in conjunction with other partners. Some of the initiatives are leadership and management strengthening, general practitioner contracting, referral system strengthening, drug and supply chain improvement, district clinical specialist team, and ward-based and school health teams.^8,10^

Universal Coverage in Tanzania and South Africa (UNITAS) was set up as one of the first systems to monitor and track UHC policy implementation in three pilot districts in South Africa.^11^ Focussing on the first NHI phase, 2011–2015 (the HSS phase), we tracked UHC policy implementation through the engagement of both policymakers and policy implementers, documenting their implementation experiences and progress towards UHC. The aim was to understand how policy–practice gaps come about in a UHC context. This study is situated in only one of the three districts, District X (name withheld to maintain anonymity). This article focusses on presenting the perspectives of that one pilot district (*n* = 71 key informants) in response to the research question ‘what do you think needs to happen for the current UHC policies to be implemented successfully, and why?’

## Methods

### Study design and theory of change

**FIGURE 1:**
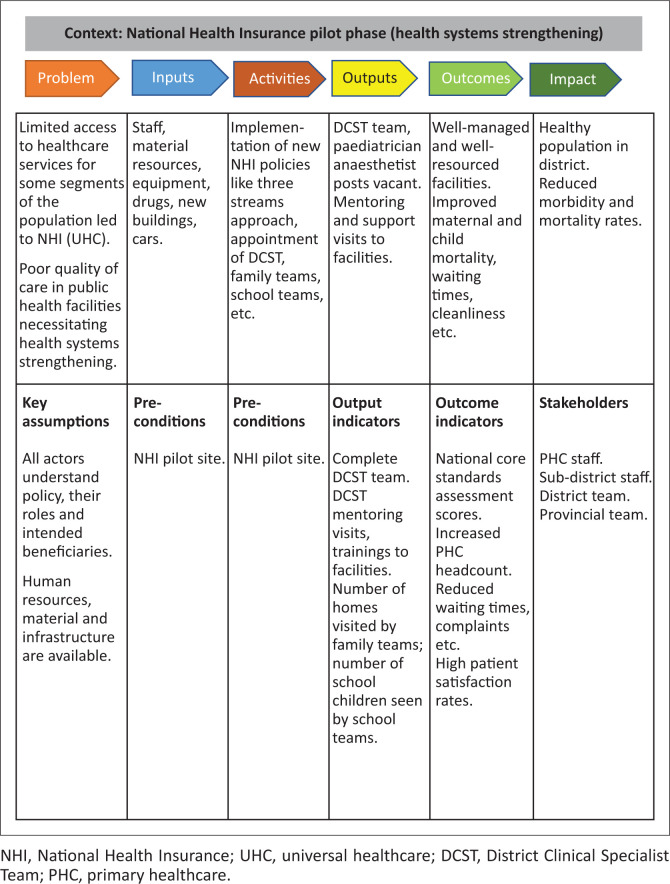
Theory of change for the National Health Insurance pilot district.

A qualitative case study design utilising a theory of change (TOC) approach was employed. A case study design is an empirical inquiry that investigates a phenomenon within its real-life context.^12^ A TOC explains how change happens.^13^ Theory of change allowed us to engage with both policymakers and implementers, understanding from their perspective how UHC policies were being implemented. It took into account the assumptions they had, their understanding of policy, beneficiaries and activities they had planned to reach policy goals. Thus, this approach facilitated a deeper and broader understanding of UHC policy implementation experience across levels in the health system. Figure 1 illustrates the TOC approach.

### Selection of the case (district)

Ten pilot districts were selected as NHI pilot sites by DoH, based on poor performance on key health indicators like high maternal and child mortality rates.^14^ Universal Coverage in Tanzania and South Africa purposively selected 3 out of the 10 pilot districts. District X was conveniently selected as the only pilot district in the province at the time. Managerial support and willingness to participate in the study also guided the site selection.

### Description of case setting

The district was run by a district health team, headed by a district manager. The district manager was supported by programme managers, PHC supervisors and sub-district managers to provide support to health facilities. The district manager reported to provincial authorities, who in turn reported to national authorities. The study took place over a period of 5 years. This was in a context of high staff turnover. One drawback, however, was that not all actors could be interviewed during all three phases as some had transferred or resigned, and a few had even died.

### Selection of respondents

Key informants included provincial actors (policymakers), where the task of operationalising NHI reforms had been assigned,^15^ and district, sub-district and PHC facility actors (policy implementers). Purposive sampling of actors at provincial and district levels was based on their knowledge of and involvement in NHI activities, their availability for interviews and willingness to participate. From district to PHC facility level, all actors were involved in NHI policy implementation, and the district and sub-district managers further assisted in the purposive selection of these key informants. Senior management, doctors and nurses from 1 district hospital, 2 community health centres and 10 PHC facilities (randomly selected after separating rural and urban facilities to ensure a balanced representation) were involved in the study. No patients were involved because their role in policy implementation is limited.

### Data collection

Qualitative data were collected during three phases: 2011–2012 (contextual mapping), 2013–2014 (Phase 1) and 2015 (Phase 2). In-depth face-to-face interviews were conducted with participants using a TOC interview guide adapted for each phase (Appendix 1). This was an iterative process of data collection and engagement with actors from contextual mapping through rounds 1 and 2. The interviews took place in departmental offices where the actors worked, at times suitable and agreed to by the participants, lasting for 2–3 hours. Two researchers, on every occasion, conducted the interviews in English. All participants were qualified professionals who understood English well. All interviews were audio-recorded. All participants gave informed and signed consent and were free to withdraw from the study at any time. Contextual mapping was carried out before the roll-out of NHI policies (2011–2012). The goal of this phase was to assess the readiness of the district to roll out NHI policies. The first round (2013–2014) interviews involved actors from province to PHC facility level. Interviews were conducted approximately one year after NHI policy roll-out, and the goal of Round 1 interviews was to elicit the experiences of policymakers and implementers one year into policy implementation. The second round was carried out in 2015. The research took place in a context of provincial moratoria on human resource recruitment; hence, there was considerable staff turnover and human resource shortages.^15^ During this round, a new provincial NHI actor was interviewed. She herself was already on her way out, as she had just resigned. Most of the district actors from the first round were interviewed, excluding one manager who had resigned and a senior one who had no time. The goal of Round 2 was to elicit from actors what they had achieved in terms of NHI policy implementation during this period. We explored with each participant what had transpired since our last visit and what the participant had achieved in terms of the activities they had planned to carry out. If they were successful, we looked for factors that facilitated implementation, and if they failed to carry out the planned activities, we also looked for factors that hindered the implementation. We concluded the second round interviews by asking all participants the following questions.

### Central question

‘What do you think needs to happen for the current UHC policies to be implemented successfully, and why? Is it with regard to information, power, interactions and motivation, resources or other factors?

#### Subquestion

‘Who in your opinion are the key structures/people or systems that are in place or need to be put in place to make these UHC interventions work at their best and to become part of routine services?’

**FIGURE 2:**
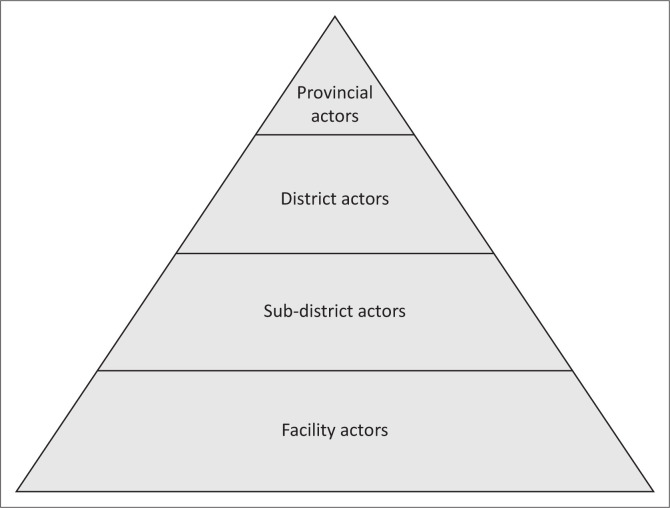
Summary of key informants and the health system level they worked on.

Figure 2 presents a summary of key informants and the health system level they worked on.

### Contextual interaction theory: A conceptual framework

The complexity of the policy implementation process has challenged researchers to develop theories and models, although with a limited number of explanatory variables that predict how and under what conditions policies are implemented.^16,17^ Implementation is far too complex to be accounted for by a single theory;^18^ at the same time, a theory or model provides a framework for systematically identifying and reporting factors that implementers perceive as affecting implementation.^17^ We identified contextual interaction theory (CIT),^19^ as it provides a simple, empirically tested framework for identifying barriers within an implementation network. The basic CIT assumption is that the course and outcome of the policy process depend not only on inputs but more crucially on the characteristics of the actors involved, particularly their motivation, information, power, resources and interactions.^20^ All other factors that influence the process do so because and insofar as they influence the characteristics of the actors involved. The theory does not deny the value of the multiplicity of possible factors but claims that, theoretically, their influence can be best understood by assessing their impact on the motivation, information, power, resources and interactions of the actors involved.^19^ Another key assumption of CIT is that factors influencing implementation are interactive. The influence of any factor, whether positive or negative, depends on the particular context, structural and outer.^20^ Based on the amount of information the actors have regarding policy, their level of motivation, the amount of resources they have to implement and the amount of power they have to mobilise the needed resources, as well as the various interactions among these factors, a policy can be successfully implemented. In this study we analysed how much information, motivation and power and how many resources they had with regard to the UHC policies they were tasked to implement. We also analysed the data inductively.

### Data analysis

All interview recordings were transcribed into Word documents. A deductive and inductive approach was utilised for data analysis. The CIT framework was used as a starting point for data extraction, allowing for new themes to be developed inductively, following Braun et al.’s approach.^21^ The analysis followed an iterative process of familiarisation with data, coding, creating and reviewing themes as well defining them whilst avoiding over-condensed data,^21^ which can lead to loss of content or context.^22^

Data management and coding were done using MAXQDA 2018 software program. Trustworthiness criteria were used to evaluate the rigour of this study.^23^ The trustworthiness concepts included dependability, credibility, confirmability and transferability. To ensure dependability, we described the data collection process in detail, and two researchers experienced in qualitative methods kept reflexive individual journals throughout the data collection and analysis. Debriefing after interviews was done daily in the field. The two researchers further analysed the data independently before reaching consensus under the supervision of experienced qualitative researchers. To ensure confirmability, the findings were discussed with supervisors experienced in the field, and their responses were incorporated. To enhance transferability, the participants, context and process of analysis have been described in detail.^23^ Data source triangulation was achieved using actors from different levels of the health system, and interviews continued until data saturation was reached.^24^

**TABLE 1:** Overview of key informants, research phase, role and where they worked (health system level).

Health system level	Role	Contextual mapping	Round 1	Round 2	Total
Provincial	Policymaker – making sure NHI policies are carried out	1	1	1	3
District	Policy implementers ranging from district manager, programme managers, district clinical specialist team and emergency rescue service manager to PHC supervisors with policy implementation responsibilities including the PHC supervision manual	1	5	4	10
Sub-district	Policy implementers at sub-district level including CEO managers, nurses and doctors implementing policies aimed at UHC as well as providing direct patient care	3	12	8	23
PHC facility	Policy implementers including operational managers and staff in PHC facilities implementing policies aimed at UHC as well as providing direct patient care	-	19	16	35

**Total**		**5**	**37**	**29**	**71**

NHI, National Health Insurance; PHC, primary healthcare; UHC, universal health coverage; CEO, chief executive officer.

Seventy-one participants involved in UHC policy implementation were interviewed. There was consensus amongst all actors, from provincial to facility level, that the current health system was set up at a different time and that the structures and systems needed transformation. Six main themes emerged from a thematic analysis of the responses given, as presented below.

The findings are firstly presented according to the CIT central tenets (information, motivation, power, resources and interactions), followed by presentation of the inductive themes. Both deductive and inductive themes were then categorised into six themes: make PHC work, transform policy development, transform policy implementation, establish public–private partnerships, transform systems and processes and adopt a systems lens.

### Deductive themes

#### Contextual interaction theory Tenet 1: Information

*‘Involve us’*: ‘Information’ refers to information about the policy itself as well as knowledge of the policy and who it is supposed to benefit. The understanding of what the NHI was, and what was improved as one went up the health system ladder, at provincial and district levels. At facility level, most of the actors viewed NHI as a novel initiative and were initially excited about it during Round 1, but this excitement could not be felt anymore in the second phase. Lack of resources to transform policy into action was a major stumbling block. The top-down nature of NHI policies further affected their motivation, as some policies were found not relevant by some facilities, and necessary equipment like vaccine fridges were not supplied. Some actors revealed the following:

‘Okay, the first thing is that consultation, I think we must be consulted, number one. Number two, they must see before the policies are finalised how much resources you need to implement this policy. And how much is available. So, considering those first, then they should finalise the policy and start implementation. Just signing policy is not enough to have it implemented and involve stakeholders in every sector, because policies must be relevant for us to implement.’ (Sub-district actor, Round 1)

#### Contextual interaction theory Tenet 2: Motivation

Apart from resources, motivation was revealed as being affected by multiple meetings that often took place without notification. Many actors cited being summoned to the district for meetings, taking them away from work and delaying policy implementation. To combat that, NHI progress meetings on the go or managing by walking about were suggested. Facility actors expected that supervisors would get to know the real issues affecting policy implementation in facilities, first-hand, that way. Seeing broken and leaking toilets might spur supervisors into action as compared to receiving a report. It is the sense of urgency that the implementing actors would like to see.

#### Contextual interaction theory Tenet 3: Power and decentralisation

The lack of power to take decisive actions, for example, to hire personnel or get resources, was cited as a huge stumbling block in policy implementation. The provincial actors agreed to a certain extent by saying that decentralisation was the way, but they also had some reservations. District, sub-district and facility actors, on the other hand, unanimously cited the need to transform the system by bringing power to the front line. Facility actors revealed that when they came up with initiatives, their supervisors did not support them. The health system design seems to stifle bottom-up innovation. There is a link between lack of power, top-down directives, bottom-up initiatives that are ignored and motivation. At another level, the tension and ambiguity between provincialisation and the district health system (DHS) could be felt:

‘So, decentralisation, yes of course it’s the way to go. A very critical starting point, but also putting then the systems that ensure that the decentralised functions, the managers are held to account. But also appreciate, what are they contributing to the bigger scheme of things. To decentralise to somebody who just enjoys the powers to be big boss, to be called a boss without using those powers that have been delegated to improve the bigger picture of things in the province. Again, it’s a waste of time and resources.’ (Provincial actor, Round 1)

‘Yes, give us the money. Give us all the powers and let us do the things ourselves.’ (Sub-district actor, Round 1)

‘The other thing that is happening to us is that our anaesthetic component no longer exists on our new structure. The district package of service does not require an anaesthetist because anyone can do local anaesthetic for a Caesar. For that kind of surgery. But because we have an influx of all of these cases, we still have anaesthetists. It is dictated by the public need. Now that needs to be brought into the minds of our principals. I have said that at meetings.’ (Sub-district actor, Round 2)

#### Contextual interaction theory Tenet 4: Resources (problem-solving and dealing with root causes, stocktaking of resources and the six building blocks before policy roll-out)

Resource shortages ranging from human, material and equipment to infrastructure were cited as challenges throughout Phase 2. The basics cited as missing included inputs such as drug shortages, processes like communication, motivation and service delivery interface challenges caused by these. Many actors cited being under pressure because of staff shortages, which in turn led to long waiting lines. Some selected facilities got new NHI buildings and were able to implement new policies like streams approach. This demonstrated that when supplied with resources, the actors were motivated to implement policies. Leadership that ensures availability of resources, problem-solving and dealing with the root causes of chronic shortages of supplies could facilitate UHC policy implementation. Some actors suggested stocktaking of resources that is holistic, efficient and encompasses the six health system building blocks before policy implementation begins. When orders are placed, turnaround times are not specified; some actors had not received the orders they had placed before our last visit, 2 years before. These issues – timelines and accountability – seemed to be of utmost importance to the facility actors, and they tie in well with that sense of urgency actors cited as missing. Knowing when identified problems would be resolved, by whom and when could move UHC closer:

‘Going back to the human resources issue, because sometimes your establishment may not have a post for an artisan, you know, or enough posts for a plumber, and yet you are expected to look after a whole big hospital plus the clinics attached to you, and you do not have a post for a plumber? And you get taps and toilets that break, so who is actually going to fix it for you?’ (Sub-district actor, Round 2)

#### Contextual interaction theory Tenet 5: Interactions – Supervision

Visits to facilities by PHC supervisors are scheduled once a month. All actors cited PHC supervision as inadequate and erratic but gave different reasons for that. At district level, actors cited the shortage of staff (PHC supervisors) and shortage of cars. At facility level, many actors were not aware of why the supervisors did not turn up but expressed how much they longed for such visits. Other facility actors expressed little hope, noting that PHC supervisors cite their own lack of power and that they hardly solve their challenges during such visits. At provincial level, the issue was viewed differently:

‘So, it is critical to get the right managers. Managers that are prepared to take risks. Managers that are prepared to embrace change, and if you still have the district managers who to them business is sitting in offices with air conditioners and comfy chairs, we will not get anywhere.’ (Provincial actor, Round 1)

### Inductive themes

#### Make primary healthcare work: Get the basics of primary healthcare right, including the preventive and promotive focus

All actors cited the need to have the basics in the health system right, particularly PHC. Primary healthcare is the first level of contact with the health system. The new family and school health teams are meant to redirect focus onto preventive and promotive health, which were neglected the first time PHC was adopted. District actors revealed challenges in retaining school health teams, as many actors saw no clear career prospects. A bottom-up initiative by the district team of buying trailers (small offices) for the family teams was not approved, leaving many discouraged. Many actors mentioned the shortage of cars for outreach as another stumbling block. On a system level, many actors revealed referral system challenges:

‘You see, things will change when the district gets its system right, and I told them this in the district meeting. If they want primary healthcare, you know the district health system has to work … If they get the district health system right, maybe we will improve.’ (Sub-district actor, Round 2)

‘I think the way to improve the health service is to make basics well-functioning: the PHC clinics should be fully functional – most of the clinics are under-staffed, there is no equipment. So, they should provide PHC facilities with staff, equipment and the training, those three things. Look, they are sent on training only to come back to a facility with no equipment, no staff, the clinic being served by one or two nurses … Well, you could make them 24-hour service providers, but then give them sufficient staff. If we improve PHC services, that will reduce the work at the hospital. Most of the clinics are open from 8 to 4 pm. Even that 8 to 4 pm shift is not functioning well due to shortage of staff, equipment and some clinics have no medicine. So, if primary healthcare is improved, then hospital care will also improve, because if our workload is reduced, we then can provide better care here.’ (Sub-district actor, Round 1)

#### Context-sensitivity and flexibility needed from supervisors

According to facility actors, many PHC supervisors seem to focus on ticking the supervision manual checklist, one-size-fits-all approach and fail to see their facilities in context. Challenges vary from one facility to another, with some being in rural settings and others in urban settings. Some facilities have staff shortages that are dire, whilst others lack essential equipment and infrastructure. To function effectively, they want their local needs and priorities to be considered.

#### Transform the way that policies are implemented

Actors revealed how they often find themselves between a policy dictate and a lack of resources on the ground to translate that policy into practice. At provincial level (policymaker), the availability of resources was viewed differently than at facility level. According to provincial actors, the earmarked NHI conditional grants had made resources readily available now more than ever. On the other hand, actors at district, sub-district and facility levels cited resource shortages as a major obstacle. National Health Insurance has quality requirements that facilities have to meet if they are to be credited. The conditional grant prescribed to the district what has to be done with the money, leaving no space for facilities to address their own specific facility priorities. Resultantly, some actors reported receiving linens (that they did not need) despite not having basic items like fire extinguishers or sprinklers in case of a fire in the facility.

#### Involve change agents

Most of the actors cited not being fully aware of their roles, and some described being handed files with new policies but being neither trained nor orientated on how to prepare for national core standards to date. They suggested:

‘We need change agents and quality champions. Yes, people need to be motivated to internalise these standards, but now when it comes to personal things like that, you need the person or the people to push to get that kind of internalisation. Quality is a way of life, and I think we have got the will. Yes, you need the will, but you need to also find the way, because it is greater than just the papers and the standards.’ (Sub-district actor, Round 2)

#### Public–private partnerships: Learning from the private sector

National Health Insurance has been associated with bringing private sector standards into the public health sector by many South Africans. South Africa has some of the best state-of-the-art private facilities in the world. All actors, from provincial to facility level, cited the private sector as a resource to be tapped into, where actors from the public sector could be seconded to the private sector to learn how to be customer oriented, effective and efficient. Others suggested partnerships like hospital twinning. The utility of these suggestions is yet to be explored.

#### Transform processes and systems

Actors at all levels without exception, from province to facility, pointed to the need of transforming all systems if NHI is to be realised. A provincial actor summed it all up:

‘But the question is, have we done enough in terms of transforming our health systems? Because probably or maybe I expect a manager to have undergone all the mentoring, the support, the coaching, the training, but she comes back and finds I’m still using the same tools but aiming to achieve different results. If I still have the same old same supply chain frameworks and same delegation, same protocols. Back to that same system, same approach? So, government is over-regulated. You have all [*this*] red [*tape*] that [*is*] affecting the whole of South Africa, for example, to recruit a doctor. Other private organisations go to those professional networks. You see a good CV there and invite the person for an interview, set up a panel, but here, I have to do a process 3 months long just to get the adverts out. So, those are the systemic processes that are very difficult to actually work within – major macro challenges.’ (Provincial actor, Round 1)

### Processes: Communication, supply chain, employee performance management and development system

Many actors cited the lack of a functional two-way communication system as a huge stumbling block, with motivational letters hardly responded to. Other actors suggested transforming supply chain systems by including an end user like a nurse in the supply chain department for efficiency purposes. Others clearly pointed out that supply chain processes, dysfunctional as they were, were a complex issue as many levels were involved. The process of maintenance was reported as dire. All actors recommended the transformation of the employee performance motivation and development system (EPMDS), which they cited as useless and a waste of time. Of particular importance seems to be the need for actors to be appreciated for what they are doing despite their current working conditions. The current EPMDS seems not to be doing that:

‘I really don’t know how that is going to happen. To implement NHI successfully, they need to put a whole lot of new systems in place. There are lots of systems that need to be put in place. This hospital is dilapidated. I’ve worked here for so many years; we’ve never been in the state that we are now. Never. It is going to take long to reach NHI, because it’s major structural changes, buildings, etc., new systems have to be put into place.’ (Sub-district actor, Round 2)

#### Plan for equipment servicing and have clear maintenance plans to save costs and improve outcomes

There are no clear maintenance or service plans for equipment, and many actors revealed that their blood pressure (BP) machines, for example, have never been calibrated, making them prone to giving false readings. A supply chain with clear service and maintenance plans for equipment could not only save costs but also outcomes. As one actor said:

‘So, our (equipment) break at the end so we end up having to buy new ones all the time, and it costs more money. Where we autoclave things? It’s broken, and I mean I spoke to the sister that’s working there. She said it’s broken because she couldn’t find oil to put into that machine, and the whole thing is now broken.’ (Sub-district actor, Round 2)

### Leadership is key

At provincial and district levels, all the actors reported having attended leadership training. Why this was not translating into results on the ground is not clear but could be related to delegations, as the DHS has not yet received full power. Other actors suggested the need for a new generation of leaders not managers – leaders who are motivated, who can think outside the box and so be able to motivate others. The process of how leaders are currently being developed was raised with the actors, suggesting a transformation of the way leaders are developed. The actors also recommended that relevant and context-specific leadership training and quality management should be incorporated in basic nurse and doctor training curricula. The sense of urgency, timelines and accountability again tied in with the leadership issues raised. As one actor said:

‘For everything else to work, the head must be strong, okay. Get the ones at the top to do their work, then all these below will do their work too. That is all.’ (Sub-district actor, Round 2)

‘The district team who wants us to be an NHI pilot site need to get involved from management level. They need to sit and say, these are the issues that are in the clinic. What can we do? We cannot shift the blame from municipality to DoH. It is a problem, and it needs to be sorted out so as to motivate the staff. I think at the moment no one wants to be here.’ (Facility actor, Round 2)

### Adopt a systems lens

The goal of NHI is to achieve UHC. To that end, multiple initiatives are being rolled out. Many actors found these initiatives – though interconnected and inter-related – not coordinated. Programme leaders often concentrated on having their indicators achieved, losing sight of the overall UHC goal. Many actors cited the need to transform the way programmes were operated and viewed, moving from a silo mentality to seeing wholes and the interconnectedness of programmes and activities, all aimed at achieving UHC. Many actors highlighted the need to adopt a systems thinking lens, identifying points of leverage whilst at the same time on the lookout for unintended consequences^25^ if UHC is to be achieved. Some actors suggested adapting some training programmes for relevance and efficiency, like training the staff manning the obstetric units to get skills in dealing with the essential steps in management of obstetric emergencies. Those people could be absorbed by wards, instead of sitting waiting to transport maternity patients. Emergency medical response system drivers could become physically involved in maternity wards, assisting with deliveries rather than only acting when a patient needs to be moved to a higher level of care. That point has leverage, considering the human resource challenges facilities are facing. As one actor revealed:

‘When we talk about NHI, we are talking about virtual electronic medical records (VEMR), so what is happening here … I do not know, people are so … they are one-sided, you know. When we [*are*] talking [*about*] electronic records, they just talk [*about*] electronic records and forget about Medipost, and when they [*are*] talking about Medipost they forget about the GP [*general practitioner*] contracting, you know. And then you find that people concentrate on one thing and forget about everything else. Yet all these things are supposed to go together.’ (Sub-district actor, Round 2)

### Organisational culture

Organisational culture comprises the values that guide the behaviour of members in an organisation. Some organisational culture issues were raised:

‘Then you also have what I would classify as micro challenges. There is the social culture, things that you cannot touch but things that are existing and persisting. In government, it’s business as usual. My job is secure. To get one person fired, no … that does not happen, they just get transferred out. You have to produce bibles of evidence. Different committees around [*will*] finally [*arrive*] at saying this person is not productive. So, those are [*organisational*] cultural decision, and quality tends to be a secondary issue.’ (Provincial actor, Round 1)

### Take epidemiological transitions into account

Apart from being involved in policy formulation, all the actors suggested taking epidemiological transitions into account, seeing the relationships and connections between demand and supply:

‘Okay. This thing happened, and that was before this HIV era. Previously, the clinics were working well. It was nice. They were seeing around about 7, 10, 15 patients, but today you cannot see 15 patients in the clinic. You can never see 10 patients; you see 60 and more, so that further makes you wonder, how can you provide all the particular quality of care that is supposed to be given with the same amount of resources?’ (Facility actor, Round 2)

### Streamline data collection for efficiency and maximising data use in planning and evaluation

Many actors suggested transforming the way data are collected, used and reported. They felt that reporting should be transformed to become a way of communicating with the higher authorities. One actor revealed the following:

‘You know, because a report does not just sit with us; it goes to national. And it goes to the Office of Health Standards and Compliance, so surely there must be red flagging, saying, what, you always fail because of cleaning. What is happening there? What do we need to do to support you? I would like to see that.’ (Sub-district actor, Round 2)

Table 2 provides a summary of emergent themes.

**TABLE 2:** Summary of emergent deductive and inductive themes.

Theme	Category	Unit meaning
Make PHC work	Get the PHC basics right Transform PHC supervision	Get the DHS right. Make the PHC facilities functional, with sufficient resources, staff, infrastructure and material. Get motivated supervisors who can provide regular supportive PHC supervision. Supervisors that help us find solutions to all problems faced by facilities. Be sensitive and flexible to the context and situation on the ground as needed. Higher-level support visits instead of assessments. Plan meetings ahead and deal with root causes when problem-solving. Streamline meetings and have constructive NHI progress meetings on the go. Include clear timelines in problem-solving.
Transform health policy development	Involve us in policy development Support bottom-up initiatives	Involve us before a policy is finalised to get to know the reality on the ground. Involving front-line staff ensures policy relevance. We know what facility priorities are. Staff establishment should be dictated by local needs. Involve us in streamlining activities and setting priorities.
Transform health policy implementation	Stocktaking of resources before policy roll-out Orientate us	Ensure resource availability and facility readiness to implement policy. Employ change agents.
Establish public–private partnerships	Learning from the private sector	Assess how the private sector works. Get some secondments from the private sector into the public sector for knowledge exchange.
Transform processes and systems	Over-regulation and systemic issues Micro-challenges Transform supply chain management Power and decentralisation Leadership development and quality management Communication channels Transform staff development and motivation systems	Red tape. Effective and efficient systems. Have clear turnaround times for solving problems. Have maintenance and service plans for equipment before they break. Include the end user in the supply chain, for example, a nurse. Power to the front line. Power and financial delegations to the district and facilities. Responsive leadership that solves problems. Responsive leadership that responds to motivation letters. Relevant and context-specific leadership training. Include quality management in the basic nursing curriculum. Two-way communication – listen to us too. New training that produces accountable leadership. Employee performance system that rewards staff. Staff appreciation. Care for the carers.
Adopt a systems lens	Adapt training programmes to maximise the efficient use of limited human resources Epidemiological transitions to be planned for Streamlining data Seeing the interconnectedness of activities and programmes	EMRS drivers trained to do deliveries. Resource and service adjustments to be done. Interact with and react to reports. Moving from silo mentality of ‘my programmes’ to seeing all programmes as interconnected with UHC as the goal.

NHI, National Health Insurance, DHS, district health system; PHC, primary healthcare; UHC, universal health coverage; EMRS, emergency medical response system.

## Discussion

**FIGURE 3:**
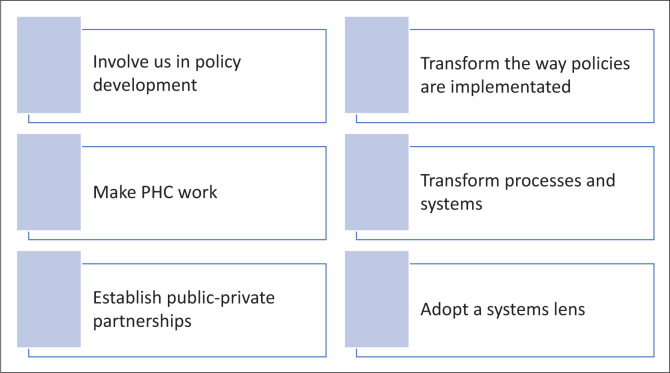
Diagrammatic summary of findings: Health system transformation rather than health system strengthening.

This study revealed that policymakers at provincial level and implementing actors at district, sub-district and facility levels view the way the current health system in South Africa is designed as a hindrance to NHI success and ultimately UHC. Our findings included make PHC work, transform policy development, transform policy implementation, establish public–private partnerships, transform systems and processes and adopt a systems lens. To strengthen means to make more effective, but actors revealed that the current systems were set up at a different socio-economic, demographic and epidemiological time. Even if they are made stronger, they have become obsolete. Concurring with our findings, the OHSC carried out audits to assess HSS activities (National Core Standards). Six priority areas were assessed, namely, improving cleanliness, reducing waiting times, improving patient safety, preventing acquired infections, improving staff attitudes and ensuring availability of medicines.^26^ Only 6% of the public health facilities met the pass mark (70%) as of March 2016.^14^ The HSS initiatives, although underway, are producing fewer results than expected. The inability of the health system to effectively implement the six health system building blocks has been identified as one of the UHC stumbling blocks, supporting our findings.^2^ Service delivery models have outlived their usefulness in meeting the changing demands of the population (see Figure 3 for a diagrammatic summary).^6^

Make PHC work first, the actors said unanimously. The basics cited as missing are in line with those described in the Primary Health Care Performance Initiative (PHCPI).^27^ Inputs (particularly human and material resources), processes (like the supply chain) and the importance of context were cited by all actors as affecting service delivery and consequently outcomes like responsiveness.^27^ What is being done currently under the HSS banner seems not to be producing the desired results. This is the reason that implementing actors are calling for health systems transformation, rather than HSS, for NHI to work. The Department of Health should consider the health system innovations suggested by the actors, like having a nurse in the supply chain.^28,29^

Primary healthcare is currently weak, underfunded, underdeveloped, ineffective and simply in crisis.^29,30^ Heavy workloads, staff and material shortages, staff–patient attitudes, long waiting times, poor supervision and support are persistent challenges.^1,2,31,32^ Our findings confirm all of these. Weak PHC was one of the top 10 global threats facing the world in 2019.^33^ We therefore recommend that DoH should go back to the drawing board and consult with and involve the implementing actors who are on the ground and are aware of the demographic, epidemiologic, technological, environmental and contextual factors that need to be taken into account to make PHC work.

The findings called for transforming the way policies are developed. Current policy development approaches are mostly top-down, with no input from actors on what works,^10,34,35^ which has contributed to the discrepancy between HSS initiatives and challenges on the ground.^10^ The actors repeatedly cited unrealistic targets set for them, without the provision of the means needed to achieve them, leading to blame when the actors failed to reach their targets. Supporting our findings, when unrealistic expectations are set by policymakers, blame is often used when expectations are not met.^30^ Early involvement of staff and taking into account the needs of the public and staff members are key to successful policy implementation.^11^ We therefore recommend that serious attention be given to involving the implementing actors in policy development as well as ensuring effective relationships between high-level policymakers and front-line workers during policy development.^36^

Public–private partnerships were suggested by many as a way the public sector could improve quality. However, very few such partnerships exist. We live in a pivotal time,^37^ as seen with coronavirus disease 2019 (COVID-19); achieving the Sustainable Development Goals, including health for all, requires fundamental changes to the way government, private and civil society work together.^37^ South Africa has a world-class private sector; hence, we recommend partnering public and private hospitals and secondment of private sector employees to governmental institutions and vice versa to facilitate innovation and organisational learning.

The lack of supportive leadership was revealed as affecting implementation. Consistent with our findings, post-1994, PHC failed in South Africa because of insufficient attention paid to implementation and weak leadership.^30^ Leadership development was revealed as not producing the required results. Supporting our findings, healthcare has failed to guide the translation of policy into health action because of a lack of leadership.^30^ Furthermore, the current health system contexts have been found to commonly encourage negative leadership practices.^38^ Poor leadership impacts negatively the staff motivation and patient care.^38^ Leadership is essential to rejuvenate and revitalise PHC.^29^ We therefore recommend that the DoH explore and develop new approaches to leadership development, utilising randomised controlled trials to explore approaches that produce leaders with exceptional skill, a sense of urgency, an attitude to serve and a sense of accountability.^39,40^

Our findings indicated that the current processes and systems needed transformation. Dysfunctional, inefficient, ineffective and outdated systems were cited as obstacles to policy implementation. These system challenges – including communication, district health system supply chain management, employee development and motivation systems and problem-solving, amongst others – have been revealed elsewhere.^30^ For healthcare services to be delivered to the desired benefit, the systems need to be functional such that health workers are available to those needing service, capable (i.e. having knowledge and skills required for that particular service), motivated to provide service and enabled (i.e. having the necessary infrastructure, equipment, drugs and other supplies).^41^ The lack of power and financial delegations were also revealed to be impeding policy implementation, concurring with research elsewhere.^35^ A lack of a supportive environment and managerial capacity in a DHS leads to service fragmentation, increased inequity and political manipulation by powerful people with vested interests.^14^ The DHS in South Africa remains poorly structured and unintegrated and is characterised by poor resources and weak managerial capacity.^32,35^ A well-functioning DHS with appropriate power and delegations is critical for UHC and PHC to succeed.^30,35^ We therefore recommend the establishment of a DHS and devolution of power.

A lack of systems thinking was cited by many as an obstacle to seeing the big picture. Many actors stated that demographic and epidemiological transitions had not been taken into account. The delivery of health services should continuously adapt and evolve according to the changing demographics and epidemiological landscape, which necessitate a health system transformation.^36^ Strong health systems are important to achieve health for all;^7,42^ however, in line with our findings, debates surrounding UHC are rarely tied to those relating to HSS or other healthcare delivery priorities on the ground.^43^ Today’s problems come from yesterday’s solutions.^25^ A weakness in one part of the system (e.g. equipment) will affect the other parts (diagnosis) and consequently the whole system.^44^ Transforming health service delivery calls for a systems lens;^6,31^ hence, we recommend systems thinking as a core component in leadership trainings. With finite resources, rationing or priority setting is needed. We therefore recommend the use of the best available evidence in doing so, as well as investing in PHC rather than on vertical programmes.

In line with our findings, a central challenge facing policymakers today is implementing health system reforms to meet the challenges of the 21st century.^36^ Health system transformation is key to deal with these implementation challenges. The World Health Organization has prioritised two key areas, namely, transforming health services to meet the growing challenges of the 21st century and moving towards UHC.^36^ Government and policymakers agree on the need to redesign the fragmented and reactive health system models now viewed as no longer fit for purpose.^36^ If the current reactive, fragmented health system model is no longer fit for purpose, can its subsystems like supply chain management, leadership development and performance management still be strengthened? The success of health system transformation rests on an understanding of both the root causes (determinants) of poor performance and the contributions of the health system itself.^6^ This can only be achieved through the engagement of health system actors involved in policymaking and implementation. Health system transformation entails (1) driving, designing and defining what makes an idea’s time come; (2) implementing and enabling the activities that support change; and (3) monitoring and feedback – assessing whether the change is working.^45^ The implementing actors are calling for this.

## Summary of recommendations

- We recommend the engagement of health system actors involved in policy implementation from policy development and implementation to evaluation.
- Primary healthcare is the first level of contact with the health system. We recommend evaluating the challenges that PHC is facing using PHCPI or a similar framework, together with the implementing actors and intentional investments into making PHC work.
- Leadership is key in policy implementation. We recommend the exploration of new leadership development approaches in context to evaluate what works.
- We recommend the establishment of DHSs and the devolution of power, as the lack of power at district level was revealed as affecting implementation.
- South Africa has changed, technologically, environmentally, socially, economically, demographically and epidemiologically – we therefore recommend going back to the drawing board to design a health system that serves the needs of today.
- A health system is a complex adaptive system with many parts. A failure in one part affects other areas and consequently the outcomes. We recommend systems thinking as a core component in nursing, doctor and leadership training.

## Limitations and strengths

This study took place in one pilot district. Qualitative studies are context specific; the findings (although transferable) may differ from studies conducted in different contexts.^46^ Finally, our study did not cover other national policies being implemented but focused only on those reforms aimed at achieving UHC. Very few systems are set up for the purpose of documenting and tracking policy implementation and monitoring in low- and middle-income countries (LMICs). This was one example of systems in South Africa to track UHC policy implementation, generating real-time evidence on why policies fail or succeed. We acknowledge that this study focused on the perceptions of actors working in the health system to gain their policy implementation experience. The views of patients, although important, were not represented, making it a limitation of the study. In this article, we managed to reveal what the actors on the front line think should be done to reach UHC. Triangulation and comparison of policy views across the different levels of the health system provided, in our minds, a balanced view of what coal face actors think needs to be done for UHC to work.

## Conclusion

With the introduction of UHC in South Africa, healthcare financing and health systems are under the spotlight. The first NHI phase (2011/2–2016/7) focused on HSS and quality improvement, and only 6% of public facilities passed muster in 2016. Our study revealed a plethora of challenges, and the implementing actors have identified a need to do things differently. What needs to happen for the current UHC policies to be implemented successfully? We started off with this question. According to the implementing actors, health system transformation rather than health system strengthening is needed to make UHC a reality. Who is listening?

## APPENDIX 1: Interview guide: Theory of change.

### Information, power and resources-related questions

Please describe your role in this facility/unit; do you play an active role in managing this facility (e.g. staffing, managing finances, power to hire or fire coordinating or reporting)?

What is your understanding of the designation of your district X (name removed for anonymity reasons) as an NHI pilot site for policies aimed at achieving UHC?

Goals of UHC particularly NHI and PHC re-engineering/key actors involved/initial communication/good for the district?

What are the policy activities that have taken place in your sub-district so far? Please tell me what your experience with each of these has been.

Let the person talk but prompt for PHC re-engineering reforms including GPs/DCSTs/management strengthening/referral system/chronic disease management/quality of care initiatives.

### Motivation-related questions

How has your facility/unit and/or you individually been prepared for working with the UHC interventions (NHI and PHC re-engineering)?

Initial roll-out/process of introducing initiatives/key actors involved in roll-out of initiatives/resources.

How have these interventions changed the way that you now work in your clinic/sub-district/programme? Please discuss whether this has been positive or challenging.

Initial adoption in the clinic/new roles for people/key changes in processes.

What do you think the desirable change (impact) of these NHI/PHC interventions is meant to bring about in your facility/district? And how?

Perceived beneficiaries/impact on the health system/what type of health and other benefits.

If you are in a management role, have you recently received any management training since 2012 –

for example, team building and interpersonal skills, communicating with others and self-awareness, financial training, human resource training, managing assets and consumables/quality assessment?

How has this training influenced the way you work and/or your relationship with others, your strengths and weaknesses?

Has the training in any way helped you implement the new UHC initiatives?

Discuss the kind of capacity building/development needs in this facility/unit.

How has the introduction of the District Clinical Specialist Team (DCST) a PHC re-engineering reform changed the way that you now work in your clinic/sub-district/programme? Please describe your experience.

Awareness/initial roll-out to DCSTs to the clinic or hospital/their role in activities/what has really worked well/thoughts on benefits for patients and staff/working as part of the team.

Has the contracting of the General Practitioners (GPs), another PHC re-engineering reform, influenced your work in any way and how? [or GP readiness/process of getting GP ready]

Awareness/Initial roll-out to facility/role in the clinic/what works well and not so well/thoughts on benefits for patients and staff/sustainability to become part of routine practice?

If your clinic is ‘GP ready’, how is the space and new equipment (if any) being used?

1. Who in your opinion are the key structure/people or systems that ARE in place or NEED to be put in place to make these UHC interventions work at their best and to become part of routine services?
2. In your experience what changes for patients have these interventions brought about so far?
3. Collective capacity of the clinic (the unit in the hospital) to function as a system and part of a system (we are trying to understand the context of the clinic):
4. Is it easy or hard to integrate new ways of working when new interventions are introduced in the facility/unit? Please explain? (Use new NHI/PHC re-engineering processes as a prompt). Ask about enablers and challenges of doing this.

### Motivation- and information-related questions

How do staff relate to each other in this facility/unit? Is there a team spirit? What enables this or what are the challenges to this?

Do you feel that this facility/unit in the hospital is capable of delivering the services that are needed for patients? Main challenges and enablers? [Prompts: space, equipment, protocols and guidelines in providing clinical services]

### Interactions

How does this facility operate as part of the sub-district (PHC system)? Do you work together with other facilities, local hospitals, sub-district offices to provide holistic quality of care for patients? [Possibly use referral system or ability to garner support from sub-district/district as prompts]

How do you use information? What information do you need to serve your clients? Where do you get this from? What works really well versus challenges with information management? (Archiving/retrieval of patient medical records)

### Motivation- and interactions-related questions

Working relationships with superiors in the clinic/unit in the hospital, type of support provided, by whom? Problem solving, performance management; tasks they do together; mentoring, support

Are there useful channels for engaging and communicating with superiors outside the facility/unit and others in the facility? How do you engage with each other?

Do you have a job description? Is there a plan in place for your continuous professional development?

What are the factors that motivate you to come to work each day?

How do you use information? What information do you need to serve your clients? Where do you get this from? What works really well versus challenges (archiving/retrieval of patient medical records)?

Any efforts made to engage communities in this clinic/CHC/hospital? How?

In your opinion what skills do managers need to help clinics/unit in the hospital/CHCs function well? Why?

Subsequent questions for rounds 1 and 2 adapted depending on previous round answers.

1. What UHC policy activities are taking place in your sub-district since 2011, or since the last interview? Please tell me what your experience with each of these has been in your role. How have these interventions changed the way that you now work in your clinic/sub-district/programme? What has been positive or challenging?
2. How has your facility/unit and/or you individually been prepared for working with the UHC interventions (NHI and PHC re-engineering), initial communication of policy, process of introducing initiatives/key actors involved in roll-out of initiatives/resources. If you are in a management role, have you recently received any management training since 2012 or since the last interview? Has the training in any way helped you implement the new UHC initiatives? How much power or delegations do you have (HR, finance, procurement and supervision)?
3. Discuss the kind of capacity building/development needs in this facility/unit? Do you feel well supported in your role? Explain why and give examples. Do you feel that this facility/unit in the hospital is capable of delivering the services that are needed for patients? Main challenges and enablers? Do you have a job description? Is there a plan in place for your continuous professional development? How is this planned and in your opinion why is this effective or not effective?
4. How have you gone about integrating the old and new ways of working when new interventions (UHC policies) were introduced in the facility/unit? What are the bottlenecks, successes and challenges you are experiencing in your current role as a UHC policymaker/implementer? Can you identify and describe an instance in the course of your duties where you or your colleagues deviated from policy? Please explain how and why this came about.
5. What do you think needs to happen for the current UHC policies to be implemented successfully and why? Is it with regard to information, power, interactions and motivation, resources, etc.? Who in your opinion are the key structures/people or systems that are in place or need to be put in place to make these UHC interventions work at their best and to become part of routine services?
6. How do staff relate to each other in this facility/unit? Is there a team spirit? What enables this or what are the challenges to this? What are the factors that motivate you to come to work each day? Working relationships with superiors in the clinic/unit in the hospital, type of support provided, by whom? (problem solving, performance management; tasks they do together; mentoring, support. In your opinion what skills do managers need to help clinics/units in the hospital/CHCs function well? Why?
7. How does this facility operate as part of the sub-district (PHC system)? Do you work together with other facilities, local hospitals and sub-district offices to provide holistic quality of care for patients? Are there useful channels for engaging and communicating with superiors outside the facility/unit and others in the facility? How do you engage with each other? Any efforts made to engage communities in this clinic/CHC/hospital? How?
8. How do you use information? What information do you need to serve your clients? Where do you get this from? What works really well versus challenges with information management?
